# Opioid prescribing to people on orthopaedic waiting lists during the covid-19 pandemic in England: retrospective cohort study using linked electronic health record data in OpenSAFELY-TPP

**DOI:** 10.1136/bmjmed-2025-001743

**Published:** 2025-12-10

**Authors:** Rose Higgins, Rebecca M Smith, Iain Dillingham, Jane Quinlan, Victoria Speed, Helen J Curtis, Christopher Wood, Milan Wiedemann, Meghna Jani, Sebastian C J Bacon, Amir Mehrkar, Ben Goldacre, OpenSAFELY Collaborative, Brian MacKenna, Andrea L Schaffer

**Affiliations:** 1 Nuffield Department of Primary Care Health Sciences, Bennett Institute for Applied Data Science, University of Oxford, Oxford, UK; 2 Oxford University Hospitals NHS Foundation Trust, Oxford, UK; 3 King’s College Hospital, King’s Thrombosis Centre, London, UK; 4 Centre for Musculoskeletal Research, Centre for Epidemiology Versus Arthritis, The University of Manchester, Manchester, UK; 5 Manchester University NHS Foundation Trust, NIHR Manchester Biomedical Research Centre, Manchester Academic Health Science Centre, Manchester, UK

**Keywords:** COVID-19, Pain management, Epidemiology

## Abstract

**Objective:**

To quantify the changes in opioid prescribing over time to a population with high rates of opioid use to understand the impact of longer elective wait times during the covid-19 pandemic.

**Design:**

With the approval of NHS England, a retrospective cohort study using linked electronic health record data in OpenSAFELY-TPP.

**Setting:**

Primary and secondary care electronic health records of people registered at general practices in England that use TPP SystmOne software, covering about 43% of the total registered population in England, linked to data from the Waiting List Minimum Dataset (WLMDS) within the OpenSAFELY-TPP platform, which is part of the NHS England OpenSAFELY covid-19 service.

**Participants:**

63 850 eligible patients on the waiting list for elective trauma procedures or orthopaedic procedures whose wait ended in admission between May 2021 and April 2022.

**Main outcome measures:**

Opioid prescribing to eligible patients before referral to the waiting list, while waiting for treatment, and after discharge from treatment. Opioids were classified based on their strength (weak, moderate, or strong opioids) and duration of action (immediate release *v* modified release opioids).

**Results:**

Of 63 850 people on elective trauma or orthopaedic waiting lists whose wait ended during the study period (median age 61 years, 54.6% female), 20.5% waited for more than 52 weeks to be admitted. In the three months before their waiting list referral date, 9890 (15.5%) participants had three or more opioid prescriptions, and 3790 (5.9%) were prescribed a strong opioid. Weekly opioid prescribing rates per 100 people on the waiting list were stable over time, with prescription rates peaking immediately after treatment and plateauing about three months after treatment. Comparing the three month period before the waiting list referral date to the period four to six months after the waiting list end date, changes in the proportion of people with three or more prescriptions for an opioid during that period were −1.6% (95% confidence interval −2.2% to −1.0%) for people on the waiting list for 18 weeks or less, −1.1% (−1.7% to −0.5%) for people waiting for 19-52 weeks, and −0.5% (−1.4% to 0.4%) for people waiting for more than 52 weeks.

**Conclusions:**

In this study, one in five people who received treatment for an elective orthopaedic procedure between May 2021 and April 2022 waited for more than one year. Nearly one in seven people were prescribed opioids long term before their referral date to the waiting list, and only small reductions in long term opioid prescribing were observed after a patient’s procedure, regardless of length of time spent on the waiting list.

**WHAT IS ALREADY KNOWN ON THIS TOPIC**

- Disruptions caused by the covid-19 pandemic increased the number of people waiting for elective care in England, and the waiting list backlog remains high even several years after the start of the pandemic
- Use of opioids may have increased for people waiting for orthopaedic treatment during this time, owing to limited access to non-drug treatments and longer waiting times requiring stronger pain relief

**WHAT THIS STUDY ADDS**

- This study quantifies the increase in patients waiting for more than 52 weeks for elective orthopaedic procedures, from 3046 patients before the covid-19 pandemic to 76 075 patients during the covid-19 pandemic, leading to prolonged preoperative exposure of patients to opioids
- Rates of long term opioid use changed little before treatment and after treatment, and longer waiting periods did not influence prescribing of opioids after treatment

**HOW THIS STUDY MIGHT AFFECT RESEARCH, PRACTICE, OR POLICY**

- Our study should encourage research to focus on the use of patient level data to monitor the clinical effect of changes in service delivery and inform evidence based policy for reducing waiting times and preventing harm to patients
- Long term opioid use is associated with poorer outcomes after treatment, and better strategies are needed to help patients reduce opioid use after receiving treatment

## Introduction

 In June 2025, 7.4 million patients were on elective waiting lists in England. This figure is a considerable increase from 4.6 million people waiting in February 2019, owing, in part, to healthcare disruptions caused by the pandemic.^1^ Some specialties are more affected than others: wait times for elective surgery, including trauma and orthopaedic procedures, fall short of the care standard of 18 weeks, with almost half of patients waiting for longer.^2^ These delays are not experienced equally, with people living in more deprived areas experiencing longer wait times.^3^

Musculoskeletal conditions are a common reason for people to receive elective orthopaedic surgery. About one third of people in the UK have a musculoskeletal condition (eg, osteoarthritis), many of whom also experience chronic pain.^4^ Two of the most common elective inpatient procedures with waiting lists are knee and hip replacement surgeries,^5 6^ with about 200 000 procedures performed in the UK in 2018.^7^ Longer waiting times may mean patients require stronger analgesia, often opioids, to manage their pain.^8^,^10^ Increased clinical use of opioids may have been exacerbated during the covid-19 pandemic, when access to non-drug treatments was limited.^11^ Major surgery (including orthopaedic surgery) is known to be associated with long term opioid use in the UK,^12^ which in turn has been associated with a higher risk of adverse outcomes after surgery in the short term and long term.^13^

To understand the effect of the longer elective waiting times experienced by patients on opioid prescribing during the covid-19 pandemic, we quantified changes in the prescribing of opioids to a population with high rates of opioid use (ie, people on a waiting list for for trauma or orthopaedic procedures). Firstly, we aimed to describe the characteristics of people on elective trauma or orthopaedic waiting lists ending in admission during the covid-19 pandemic. Secondly, we quantified how patterns of opioid prescribing to patients changed before their waiting list referral date, during their wait, and after their procedure. Finally, we established how these patterns varied by the length of time patients spent on the waiting list.

## Methods

### Context

In England, patients referred by the NHS for a physical or mental health condition have a right to start non-urgent, consultant led treatment within a maximum waiting time of 18 weeks. A patient's waiting time starts at the date of referral to treatment, and ends when a clinician decides against treatment, when the patient declines treatment, or when treatment for the referred condition starts. Prescribing for patients being referred through their primary care provider is captured in the primary care data, because this is the patient’s first point of contact with the health service for most non-urgent care.

### Monthly aggregate waiting list data

Two data sources relating to waiting lists are available: one contains person level data on waiting times, and the other contains national waiting list data grouped by specialty area and care provider. Person level waiting list data were only available between May 2021 and April 2022 at the time we performed our analysis. Therefore, to understand opioid prescribing trends before the covid-19 pandemic, we used the publicly available monthly aggregate referral-to-treatment data for patients on waiting lists, whose wait ended in a hospital admission, between January 2019 and June 2022.^14^ Referral-to-treatment waiting lists cover waits for consultant led, elective procedures^15^ (a glossary of terms is available in online supplemental box 1).

We restricted our analysis to people waiting for trauma or orthopaedic specialty procedures, because these surgeries are commonly performed to treat conditions that require pain relief.^16 17^ Trauma and orthopaedic procedures were defined using the treatment function code 110 (trauma and orthopaedic service), which includes services to treat disorders (congenital and acquired) of and injuries to the bones, joints, and their associated soft tissues, including ligaments, nerves, and muscles.^18 19^ We could not distinguish orthopaedic procedures from trauma procedures, so both types of activity were included in our analysis. However, most trauma procedures are conducted through urgent care pathways and thus were out of scope of the referral-to-treatment data.

### Person level waiting list data

#### Data source

Primary care records managed by TPP, the general practice software provider, were linked to Hospital Episodes Statistics Admitted Patient Care data, the Office for National Statistics (ONS) death data and the Waiting List Minimum Dataset through the OpenSAFELY platform, part of the NHS England OpenSAFELY covid-19 service. The ONS registered deaths dataset is a longstanding data collection, while the Waiting List Minimum Dataset is a new data collection rapidly established in response to the covid-19 pandemic and first approved in March 2021.^20^ The data were linked, stored, and analysed securely within the OpenSAFELY platform (https://opensafely.org) as part of the NHS England OpenSAFELY covid-19 service. Patient data are pseudonymised and contains information on diagnoses, drugs, and physiological characteristics. No free text data, which may contain identifiable information are included, and all analytical code is shared openly for review and reuse under the Massachusetts Institute of Technology open licence (https://github.com/opensafely/waiting-list). Detailed pseudonymised patient data are potentially reidentifiable and therefore not available for sharing. A detailed overview of OpenSAFELY is available elsewhere.^21^ The Waiting List Minimum Dataset contains weekly data for people who have been referred-to-treatment, and are currently on the waiting lists, and is subject to less validation than the aggregated monthly statistics, and therefore the data are less complete.^20^

#### Study population

We identified all patients who were registered with a general practitioner using TPP electronic health record software, and who were on a waiting list for an elective (ie, routine, non-urgent) procedure between May 2021 and April 2022. We further restricted this population to those people whose waiting list for trauma and orthopaedic procedures ended in admission only. Non-admitted pathways include outpatient procedures and people whose waiting time ended for reasons other than hospital admission (eg, the patient declined treatment, entered active monitoring, died, or the decision was made not to treat them).^15^ We could not accurately ascertain the exact reason for non-admission from the data, so we excluded data from patients whose waits did not end in an admission.

We excluded people with missing or impossible values of age or sex (<0.01%), because this category indicates poor data quality. Datapoints for extreme waiting times may be spurious outliers; therefore, to reduce their impact on the analysis, we also excluded people with waiting time values greater than the 99.9th centile (2.5 years). Finally, we excluded people who were younger than 18 years when they were referred to the waiting list, and separately, patients with a history of cancer who had received a cancer diagnosis in the five years before the waiting list’s start date, because they may be using opioids to treat cancer related pain.

We restricted people in our study population with more than one eligible pathway to only their most recently completed pathway. Participants had to be registered with their general practice from six months before their waiting list referral date to their waiting list end date to be eligible. If a patient deregistered from their practice or died during the study period, they were censored (details of inclusion criteria and exclusion criteria are in online supplemental figure 1).

#### Study measures

The referral date to a consultant led service for treatment is the start of a patient's time spent on the waiting list, and is known as the waiting list referral date in our analysis (online supplemental box 1). For patients on an admitted pathway, the end date of the wait is the date of their admission to hospital, where that admission includes first definitive treatment. We defined key variables for this population, according to the waiting list referral date: age, sex, index of multiple deprivation group (separated by deciles), and ethnic group. Missing values for index of multiple deprivation and ethnic group were categorised separately. We also identified the presence of the following health conditions in our study population: symptoms of anxiety; cardiac disease; chronic kidney disease; chronic respiratory disease; symptoms of depression; diabetes; osteoarthritis; rheumatoid arthritis; or severe mental illness (eg, bipolar disorder, schizophrenia, psychosis). Osteoarthritis and rheumatoid arthritis commonly require analgesia, and mental health problems are also associated with increased opioid use by patients.^22^

#### Opioid prescribing

We identified prescribing of all opioids for analgesia in the primary care setting, defined as those falling under the British National Formulary chapters 4.7.2 (opioid analgesics), in addition to opioids in combination with paracetamol and/or ibuprofen under chapters 4.7.1 and 10.1.1. We excluded opioids not primarily indicated for pain, such as codeine used for cough suppression (chapter 3.9.1), motility problems (chapter 1.4.2), alfentanil or fentanyl for general anaesthesia (chapter 15.1.4), and buprenorphine or methadone for opiate substitution treatment (chapter 4.10.3).

We also classified opioids based on their strength (weak, moderate, or strong opioids) and duration of action (immediate release *v* modified release opioids). Weak opioids included codeine, meptazinol, and dihydrocodeine, moderate opioids included tramadol and tapentadol, and strong opioids included all other formulations.^23^ Immediate release opioids are preferred for treatment of pain after surgery, because of the risk of harm associated with modified release formulations in people who have had surgery.^24 25^ All codelists for medicines and comorbidities in this study are available in our Github repository: https://github.com/opensafely/waiting-list.

#### Other analgesic prescribing

We identified the prescribing of other medicines to treat pain in the three months before an individual’s waiting list referral date: antidepressants (including those indicated for treatment of neuropathic pain (eg, amitriptyline, duloxetine)),^26^gabapentinoids, and non-steroidal anti-inflammatory drugs (NSAIDs).

### Analysis

We took two approaches to understand the changes in opioid prescribing to people on a waiting list during the covid-19 pandemic. Firstly, we compared opioid prescribing immediately before the patient’s waiting list referral date with opioid prescribing after the waiting list end date (ie, when they were admitted to hospital for treatment). Secondly, we looked at weekly changes in prescribing patterns throughout the study period. We did this for patterns of overall opioid prescribing, and for different types of opioid (weak, moderate, or strong opioids, and immediate release or modified release opioids). To prevent any potential disclosure of personally identifiable data, all counts are rounded to the nearest five.

#### Before-and-after analysis

We calculated the number of people with one or more opioid prescriptions and people with three or more opioid prescriptions in the three months before their waiting list referral date, and in the period four to six months after their waiting list end date. We chose to include people with three or more opioid prescriptions in the three months before referral to the waiting list, because this metric has previously been used to define long term opioid use in UK routine data.^27^

For the period after treatment, we excluded the first three months after the waiting list end date and focused on the four to six months after the waiting list end date (online supplemental figure 2). We focused on this period to exclude the time immediately after treatment, when people were using opioids temporarily to treat pain resulting from their procedure. Furthermore, Royal College of Anaesthetists guidelines state that opioid use beyond three months after surgery warrants further assessment of patients.^24^ To calculate rates of prescribing in the period after surgery, we only included people who had full follow-up (ie, who were still alive and registered with the same practice).

#### Changes over time

We quantified the opioid prescribing rate (per 100 people) for each week from 26 weeks before to a patient’s waiting list referral date, during time spent on the waiting list, and up to 52 weeks after the waiting list end date. Given the variability in waiting times, we chose to use weekly prescribing rates (instead of monthly), to make most use of all the available data. In our figures, time spent on the waiting list by patients was truncated at 52 weeks.

In each week, the numerator was the number of people prescribed an opioid in that week, and the denominator was the total number of people still on the waiting list. For instance, to calculate the opioid prescribing rate for week 18 on the waiting list, only people waiting for 18 weeks or longer were included.

To understand how prescribing patterns varied by time on the waiting list, we also stratified by waiting list duration (≤18 weeks, 19-52 weeks, and >52 weeks). These categories were chosen as a waiting time of 18 weeks or less is considered the standard waiting time for non-urgent procedures, and NHS England policy states that no one should wait for more than 52 weeks from referral to first treatment.^28^ To better visualise the non-linear changes in the prescribing rate over time, we smoothed the data using loess regression.

#### Subgroup analyses

Specific reasons why patients were on the waiting list were unavailable, so we performed two subgroup analyses. Firstly, we restricted our analysis to people with a recorded diagnosis of osteoarthritis in primary care in the past five years. Secondly, we found people who had received an orthopaedic hip or knee procedure by linking to Hospital Episode Statistics Admitted Patient Care data, and we identified them using Healthcare Resource Groups codes (online supplemental table 1).

### Patient and public involvement

Our work with the OpenSAFELY service has involved patients and the public in various ways. We developed a publicly available website that describes the platform in language suitable for a lay audience (https://opensafely.org), and we have participated in two citizen juries exploring public trust in OpenSAFELY. We co-developed an explainer video for the service (https://www.opensafely.org/about/), and recruited patient representatives, who are experts by experience, to join our OpenSAFELY Oversight Board. As part of our partnership with Understanding Patient Data, an organisation which aims to ensure data is being used in ways which are worthy of public trust, we produced lay explainers on the importance of large datasets for research, and we have presented our work at various online public engagement events to key communities (eg, Healthcare Excellence Through Technology, the Faculty of Clinical Informatics’ annual conference, NHS Assembly, and Health Data Research UK symposium). To ensure that patients’ voice are represented, we are working closely to decide on language choices with appropriate medical research charities (eg, the Association of Medical Research Charities). We will share data from and interpretation of our findings through press releases, social media channels, and plain language summaries.

## Results

### Monthly aggregate waiting list data

**Figure 1:**
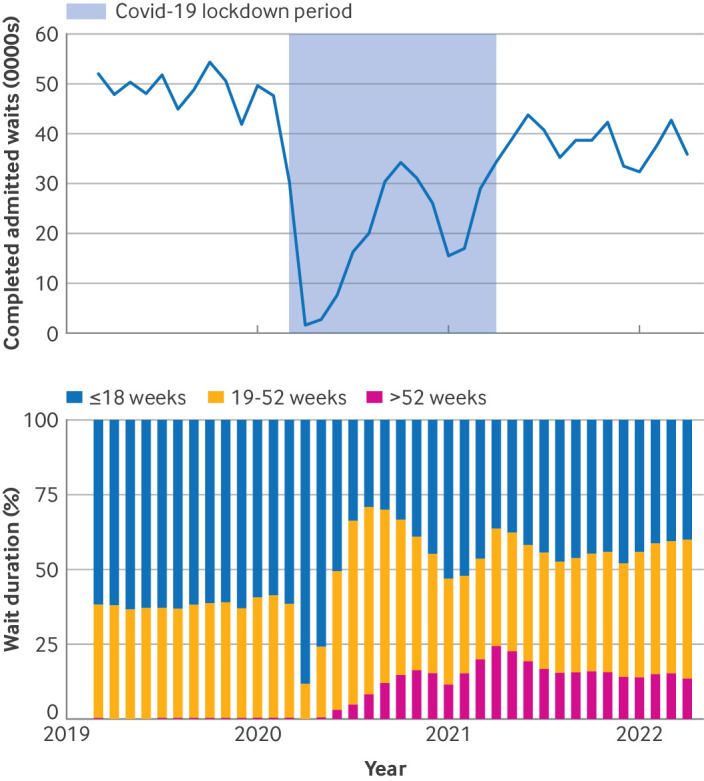
Number of completed waits for elective trauma and orthopaedic admitted procedures based on month of waiting list end date (ie, admission to hospital for treatment; top) and time on waiting list for these procedures (bottom), from January 2019 to April 2022

In the year before the covid-19 pandemic began (March 2019 to February 2020), 587 977 people were admitted for elective trauma or orthopaedic treatment, with a median of 49 253 patients admitted per month (figure 1). In this period, the overall proportion of people who waited for more than 52 weeks to be admitted to hospital was 0.5% (n=3046) (figure 1).

Following decreases in the number of patient admissions on the waiting list during the covid-19 lockdowns in England^12^ from March 2020 to April 2021, 460 830 patients were admitted for elective trauma or orthopaedic procedures from May 2021 to Apr 2022, with a median of 38 688 patients admitted per month. In total, 76 075 (16.5%) patients had waited for more than 52 weeks before being admitted for treatment, with 260 762 (56.7%) waiting for more than 18 weeks before admission.

### Person level waiting list data

In the person level waiting list data, we found nearly three million people on an referral to treatment waiting list that ended between May 2021 and April 2022. Of these, 235 540 were waiting for trauma or orthopaedic specialty treatment, 85.6% of which were elective (ie, not urgent). After restricting to people waiting for elective inpatient treatment and applying our exclusion criteria, we identified 63 850 people in our study cohort (table 1**,** online supplemental figure 1). Of these patients, 6150 (9.6%) were on more than one eligible waiting list pathway. The waiting list referral and end dates are in online supplemental figure 3. The median age was 61 years (interquartile range (IQR) 49-72 years) and 54.6% of patients were female. The median time spent on the waiting list was 26 weeks (IQR 12-47 weeks); 24 390 patients (38.2%) waited for 18 weeks or less, 26 375 (41.3%) waited for 19-52 weeks, and 13 090 (20.5%) waited more than 52 weeks for admission.

**Table 1:** Characteristics of people waiting for elective inpatient trauma or orthopaedic procedures between 1 May 2021 and 30 Apr 2022, overall and stratified by opioid prescribing to patients in the three month period before their waiting list referral date

Characteristic	Full cohort (n=63 850)	3 months before waiting list referral date		
		Not prescribed an opioid (n=43 275)	≥1 prescription (n=20 575)	≥3 prescriptions (n=9890)
**Age on waiting list referral date (years)**				
18-39	8915 (14.0)	7545 (17.4)	1380 (6.7)	510 (5.2)
40-49	7165 (11.2)	5180 (12.0)	1985 (9.6)	940 (9.5)
50-59	13 530 (21.2)	9105 (21.0)	4450 (21.6)	2185 (22.1)
60-69	15 085 (23.7)	9495 (21.9)	5600 (27.2)	2815 (28.5)
70-79	14 180 (22.2)	8985 (20.8)	5215 (25.3)	2495 (25.2)
≥80	4905 (7.7)	2965 (6.9)	1940 (9.4)	940 (9.5)
**Sex**				
Female	34 820 (54.6)	22 295 (51.5)	12 565 (61.1)	6195 (62.6)
Male	28 960 (45.4)	20 980 (48.5)	8005 (38.9)	3695 (37.4)
**Index of multiple deprivation group (separated by decile)**				
1 (most deprived)	5310 (8.3)	3105 (7.2)	2205 (10.7)	1210 (12.2)
2	5790 (9.1)	3550 (8.2)	2245 (10.9)	1200 (12.1)
3	5670 (8.9)	3590 (8.3)	2080 (10.1)	1075 (10.9)
4	6260 (9.8)	4140 (9.6)	2125 (10.3)	1040 (10.5)
5	6730 (10.6)	4565 (10.5)	2170 (10.5)	1035 (10.5)
6	7500 (11.8)	5210 (12)	2300 (11.2)	1050 (10.6)
7	6940 (10.9)	4870 (11.3)	2080 (10.1)	970 (9.8)
8	6495 (10.2)	4630 (10.7)	1875 (9.1)	825 (8.3)
9	6550 (10.3)	4795 (11.1)	1760 (8.6)	760 (7.7)
10 (least deprived)	5345 (8.4)	3985 (9.2)	1365 (6.6)	545 (5.5)
Missing	1195 (1.9)	830 (1.9)	365 (1.8)	180 (1.8)
**Ethnic group**				
White	46 990 (73.7)	31 580 (73)	15 470 (75.2)	7560 (76.4)
Black	1600 (2.5)	1170 (2.7)	435 (2.1)	160 (1.6)
South Asian	615 (1.0)	435 (1)	180 (0.9)	60 (0.6)
Mixed	345 (0.5)	255 (0.6)	90 (0.4)	30 (0.3)
Other	345 (0.5)	275 (0.6)	70 (0.3)	25 (0.3)
Unknown	13 875 (21.8)	9560 (22.1)	4325^21^	2060 (20.8)
**Conditions recorded in primary care in past five years**				
Anxiety (symptoms or diagnosis)	7730 (12.1)	5015 (11.6)	2730 (13.3)	1385 (14.0)
Cardiac disease	6880 (10.8)	4010 (9.3)	2880 (14)	1545 (15.6)
Chronic kidney disease	2255 (3.5)	1350 (3.1)	910 (4.4)	470 (4.8)
Chronic respiratory disease	2780 (4.4)	1405 (3.2)	1385 (6.7)	815 (8.2)
Depression (symptoms or diagnosis)	8410 (13.2)	5060 (11.7)	3370 (16.4)	1820 (18.4)
Diabetes	5975 (9.4)	3350 (7.7)	2625 (12.8)	1410 (14.3)
Osteoarthritis	24 225 (38.0)	13 910 (32.1)	10 345 (50.3)	5160 (52.2)
Rheumatoid arthritis	1255 (2.0)	630 (1.5)	630 (3.1)	365 (3.7)
**Prescribing three months before waiting list referral date**				
Antidepressants				
≥1 prescription	17 180 (26.9)	8385 (19.4)	8795 (42.7)	5120 (51.8)
≥3 prescriptions	9760 (15.3)	4195 (9.7)	5565 (27.0)	3850 (38.9)
Amitriptyline/duloxetine				
≥1 prescription	7650 (12.0)	3100 (7.2)	4550 (22.1)	2760 (27.9)
≥3 prescriptions	3720 (5.8)	1255 (2.9)	2465 (12.0)	1860 (18.8)
Gabapentinoids				
≥1 prescription	6335 (9.9)	2250 (5.2)	4085 (19.9)	2805 (28.4)
≥3 prescriptions	4020 (6.3)	1230 (2.8)	2790 (13.6)	2215 (22.4)
Non-steroidal anti-inflammatory drugs				
≥1 prescription	10 275 (16.1)	4975 (11.5)	5300 (25.8)	2605 (26.3)
≥3 prescriptions	3205 (5.0)	1185 (2.7)	2020 (9.8)	1425 (14.4)
**Died within six months of waiting list end date**				
No (%)	280 (0.4)	135 (0.3)	180 (0.9)	85 (0.9)

Data are number (%) of people.

#### Before-and-after analysis

About one third of participants (n=20 575, 32.2%) were prescribed an opioid in the three months before their waiting list referral date (table 1), and 15.5% (n=9890) had three or more opioid prescriptions in that time. Immediate release opioids were more commonly prescribed—given to 29.7% of the study population—than modified release (5.2%; table 2). Weak opioids were prescribed to 23.7% of participants, compared with 6.6% of participants being prescribed a moderate opioid and 5.9% a strong opioid. People with three or more opioid prescriptions were more likely to be older, to be female, and to be living in more deprived areas than those not prescribed opioids. They were also more likely to have a diagnosis of osteoarthritis than those not prescribed opioids (52.2% *v* 32.1%; table 1) and were much more likely to be prescribed antidepressants (51.8% *v* 19.4%; table 1) and other analgesic medicines, including gabapentinoids (28.4% *v* 5.2%; table 1) and non-steroidal anti-inflammatory drugs (26.3% *v* 11.5%; table 1).

**Table 2:** Opioid prescribing before and after time spent on waiting list among 63 850 people waiting for elective inpatient trauma or orthopaedic procedures, by time on waiting list (≤18 weeks, 19-52 weeks, >52 weeks)

Prescribing groups	≥1 opioid prescription			≥3 opioid prescriptions		
	Pre-waiting list	Post-waiting list	Difference (%; 95% CI)	Pre-waiting list	Post-waiting list	Difference (%; 95% CI)
**Full cohort**						
Any opioid	20 575 (32.2)	16 220 (26.9)	−5.4 (−5.8 to −4.8)	9890 (15.5)	8650 (14.3)	−1.2 (−1.6 to −0.8)
Immediate release opioid	18 970 (29.7)	14 650 (24.3)	−5.4 (−5.9 to −4.9)	8135 (12.7)	7010 (11.6)	−1.1 (−2.1 to −0.2)
Modified release opioid	3330 (5.2)	3055 (5.1)	−0.2 (−0.4 to 0.1)	2300 (3.6)	2245 (3.7)	0.1 (−0.5 to 0.7)
Weak opioid	15 150 (23.7)	11 195 (18.5)	−5.2 (−5.8 to −4.7)	5655 (8.9)	4865 (8.1)	−0.8 (−2.4 to 0.8)
Moderate opioid	4210 (6.6)	3370 (5.6)	−1.0 (−1.3 to −0.7)	2000 (3.1)	1800 (3.0)	−0.2 (−0.8 to 0.5)
Strong opioid	3790 (5.9)	3480 (5.8)	−0.2 (−0.4 to 0.1)	2465 (3.9)	2395 (4.0)	0.001 (−0.1 to 0.3)
**≤18 weeks on waiting list**						
Any opioid	7660 (31.4)	5925 (25.8)	−5.6 (−6.4 to −4.8)	3785 (15.5)	3195 (13.9)	−1.6 (−2.2 to −1.0)
Immediate release opioid	7035 (28.8)	5340 (23.2)	−5.6 (−6.4 to −4.8)	3110 (12.8)	2565 (11.2)	−1.6 (−2.2 to −1.0)
Modified release opioid	1315 (5.4)	1160 (5)	−0.4 (−0.8 to 0.0)	920 (3.8)	865 (3.8)	0.0 (−0.3 to 0.3)
Weak opioid	5485 (22.5)	4030 (17.5)	−5.0 (−5.7 to −4.3)	2085 (8.6)	1760 (7.7)	−0.9 (−1.4 to −0.4)
Moderate opioid	1605 (6.6)	1280 (5.6)	−1 (−1.4 to −0.6)	790 (3.2)	685 (3.0)	−0.2 (−0.5 to 0.1)
Strong opioid	1590 (6.5)	1335 (5.8)	−0.7 (−1.1 to −0.3)	1020 (4.2)	930 (4.0)	−0.2 (−0.6 to 0.2)
**19-52 weeks on waiting list**						
Any opioid	8540 (32.4)	6695 (26.8)	−5.6 (-6.4 to −4.8)	3985 (15.1)	3505 (14)	−1.1 (−1.7 to −0.5)
Immediate release opioid	7935 (30.1)	6070 (24.3)	−5.8 (−6.6 to −5.0)	3315 (12.6)	2875 (11.5)	−1.1 (−1.7 to −0.5)
Modified release opioid	1280 (4.9)	1190 (4.8)	−0.1 (−0.5 to 0.3)	875 (3.3)	865 (3.5)	0.2 (−0.1 to 0.5)
Weak opioid	6445 (24.4)	4705 (18.8)	−5.6 (−6.3 to −4.9)	2380 (9)	2025 (8.1)	−0.9 (−1.4 to −0.4)
Moderate opioid	1680 (6.4)	1325 (5.3)	−1.1 (−1.5 to −0.7)	765 (2.9)	700 (2.8)	−0.1 (−0.4 to 0.2)
Strong opioid	1425 (5.4)	1360 (5.4)	0 (−0.4 to 0.4)	930 (3.5)	920 (3.7)	0.2 (−0.1 to 0.5)
**>52 weeks on waiting list**						
Any opioid	4380 (33.5)	3610 (29.1)	−4.4 (−5.5 to −3.3)	2120 (16.2)	1945 (15.7)	−0.5 (−1.4 to 0.4)
Immediate release opioid	4000 (30.6)	3240 (26.2)	−4.4 (−5.5 to −3.3)	1710 (13.1)	1570 (12.7)	−0.4 (−1.2 to 0.4)
Modified release opioid	735 (5.6)	705 (5.7)	0.1 (−0.5 to 0.7)	505 (3.9)	515 (4.2)	0.3 (−0.2 to 0.8)
Weak opioid	3220 (24.6)	2460 (19.9)	−4.7 (−5.7 to −3.7)	1190 (9.1)	1080 (8.7)	−0.4 (−1.1 to 0.3)
Moderate opioid	925 (7.1)	765 (6.2)	−0.9 (−1.5 to −0.3)	450 (3.4)	410 (3.3)	−0.1 (−0.5 to 0.3)
Strong opioid	775 (5.9)	790 (6.4)	0.5 (−0.1 to 1.1)	510 (3.9)	545 (4.4)	0.5 (0.0 to 1.0)

Data are number (%) of people unless stated otherwise. Pre-waiting list is three months before referral date, and post-waiting list is months four to six after waiting list end date. Post-waiting list excludes participants lost to follow up.

CI, confidence interval.

We compared data from the four to six months after the patient received treatment with data from the three months before their waiting list referral date, and found that fewer people received an opioid prescription after treatment (−5.4%, 95% confidence interval (CI) −5.8% to −4.8%). We also found slight reductions in the proportion of people prescribed immediate release opioids (−5.4%, 95% CI −5.9% to −4.9%) but prescribing of modified release opioids did not change (−0.2%, 95% CI −0.4% to 0.1%). Similarly, fewer people were prescribed weak opioids (−5.2%, 95% CI −5.8% to −4.7%) but only slight changes were observed for moderate and strong opioids. We saw substantial variation in opioid prescribing by age, sex, and index of multiple deprivation group (table 1).

The proportion of people with three or more opioid prescriptions decreased from 15.5% in the three months before being referred to the waiting list to 14.3% after the patient recieved treatment (−1.2%, 95% CI −1.6% to −0.8%) (table 2). When stratified by time on the waiting list, we observed similar patterns. We also found slight variation in the opioid prescribing rate before a patient was referred to the waiting list, ranging from 31.4% for one or more opioid prescriptions to 15.5% for three or more opioid prescriptions, for people waiting for 18 weeks or less; and ranging from 33.5% to 16.2%, respectively, for people waiting for more than 52 weeks. During the four to six months after treatment, the corresponding values ranged from 25.8% to 13.9% for people waiting for 18 weeks or less; and ranged from 29.1% to 15.7%, for people waiting for more than 52 weeks (table 2). The reduction in proportion of people with three or more opioid prescriptions ranged from −0.5% for people waiting for more than 52 weeks to −1.6% for people waiting for 18 weeks or less.

#### Changes in weekly opioid prescribing over time

**Figure 2:**
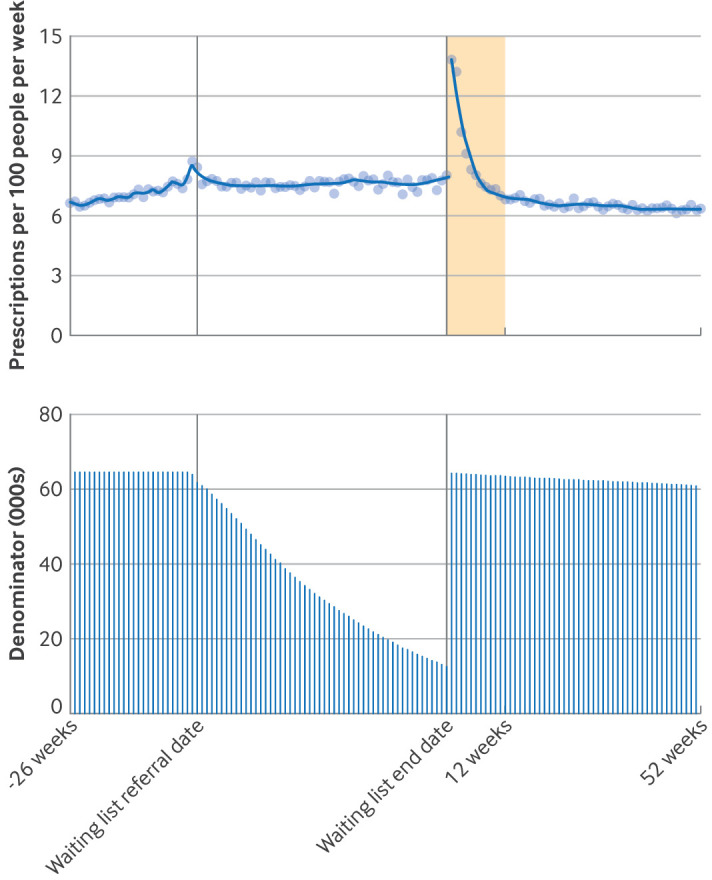
Number of opioid prescriptions per week per 100 people waiting for elective trauma and orthopaedic admitted procedures, from 26 weeks before waiting list referral date, during the period on the waiting list, and up to 52 weeks after waiting list end date (date of admission for treatment). Dots=observed values; solid lines=values predicted from loess regression model; shaded area=three months after treatment. During the waiting list period, the denominator includes everyone who was still on the waiting list at the end of each week. During the waiting list period and the period after treatment, the denominator excludes people who died or who deregistered from their general practice

In the six months before a patient’s waiting list referral date, the median number of prescriptions per week was 6.6 per 100 people at 26 weeks before the waiting list referral date, increasing to 8.7 weekly prescriptions per 100 people the week immediately preceding the referral date (figure 2). During the time spent on the waiting list, the median weekly prescribing rate was 7.7 per 100 patients (IQR 7.5-7.9).

**Figure 3:**
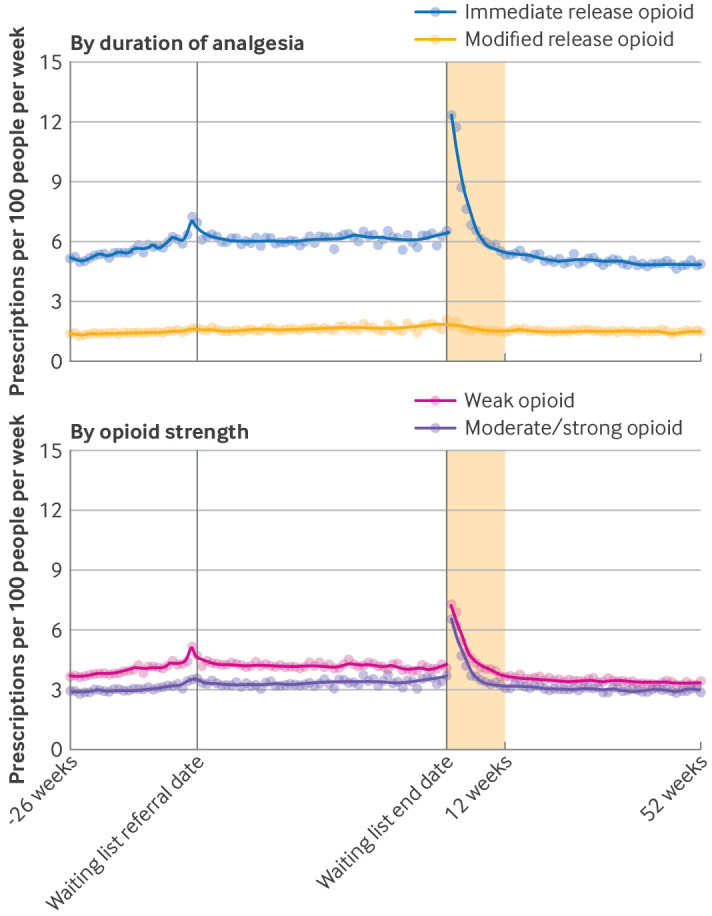
Number of opioid prescriptions by opioid type per week per 100 people waiting for elective trauma and orthopaedic admitted procedures, in the 26 weeks before the waiting list referral date, during the waiting list, and up to 52 weeks after waiting list end date (date of admission for treatment). Dots=observed values, solid lines=values predicted from loess regression model; shaded area=three months after treatment. During the waiting list period, the denominator includes everyone who was still on the waiting list at the end of each week. During the waiting list period and after treatment periods, the denominator excludes people who died or who deregistered from their general practice

We found a large increase in opioid prescribing immediately after treatment (13.8 weekly prescriptions per 100 people), which stabilised after about three months. From three months to one year after a patient was admitted for treatment, the median number of prescriptions was 6.5 per 100 people per week (figure 2). When stratified by opioid type, the largest changes were for immediate release opioids (figure 3).

**Figure 4:**
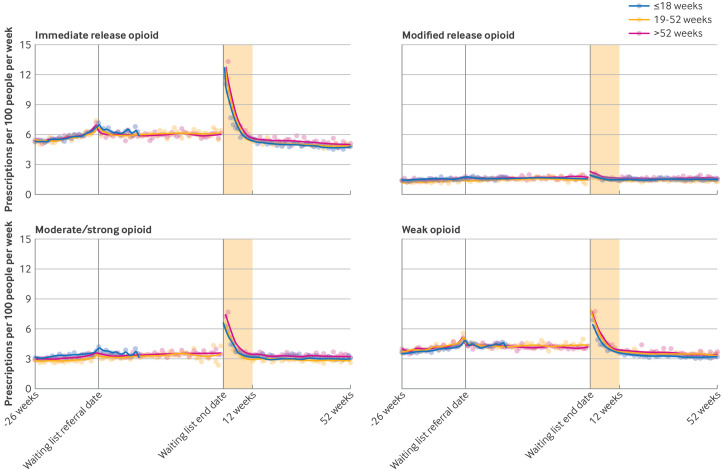
Number of opioid prescriptions per week per 100 people waiting for elective trauma and orthopaedic admitted procedures, in the 26 weeks before the waiting list referral date, during the waiting list, and 52 weeks after waiting list end date (date of admission for treatment), stratified by time spent on waiting list and by opioid type. Dots=observed values; solid lines=values predicted from loess regression model; shaded area=three months after admission. During the waiting list period, the denominator includes everyone who was still on the waiting list at the end of each week. During the waiting list period and after treatment periods, the denominator excludes people who died or who deregistered from their general practice

Stratified by time on the waiting list, in the six months before the referral date to waiting list, the median prescribing rates ranged from 6.8 to 7.2 per 100 people per week. In the four to six months after the waiting list end date, the median weekly prescribing rates ranged from 6.4 to 6.9 per 100 people (figure 4). We observed similar patterns when we analysed by total time a patient spent on the waiting list. In all groups, prescribing stabilised after about three months.

### Subgroup analyses

In our sensitivity analyses, 24 255 people had a diagnosis of osteoarthritis, 10 160 people had a hip procedure, and 14 975 had a knee procedure. People who had an inpatient knee procedure were younger and more likely to be men than those who has a hip procedure or a diagnosis of osteoarthritis (online supplemental table 2). Before the waiting list referral date, people who had undergone a knee procedure comprised a smaller fraction of people with three or more opioid prescriptions compared to those with a diagnosis of osteoarthritis and those who had undergone a hip procedure (14.8 *v* 21.3% and 21.5%, respectively). People with a hip procedure had the greatest reduction in opioid prescribing, when comparing before being referred to the waiting list and after leaving the waiting list: rates changed from 44.5% to 30.2% for people with one or more prescriptions, and 21.5% to 15.8% for people with three or more prescriptions (online supplemental table 3). These patterns were the same in the plots showing changes in weekly prescribing over time (online supplemental figure 4-6).

## Discussion

### Summary

Before the covid-19 pandemic, waiting times of more than 52 weeks for elective trauma or orthopaedic procedures were very rare, with 0.5% of people waiting for over 52 weeks. However, during the covid-19 pandemic, our previously small study population increased from 3046 people to 76 075 people, and more than half of all waits for elective trauma or orthopaedic surgeries exceeded the 18 week NHS treatment target. In our study population, about one third of patients were prescribed an opioid in the three months leading up to their waiting list referral date, and nearly one in seven had three or more prescriptions (which is sometimes considered long term use)^27^ with minimal changes to this prescribing rate after patients recieved treatment. We found similar patterns of opioid prescribing after surgery regardless of the time spent by patients on the waiting list.

### Findings in context

Other studies analysing changes in opioid prescribing during the covid-19 pandemic found no sustained increases in rates of opioid prescribing, either in the general population or in people with musculoskeletal diseases.^29 30^ However, a Scottish study investigating opioid use in people receiving hip and knee arthroplasty found that rates of opioid use after surgery was higher during the covid-19 pandemic than in historic controls.^8^ While we observed only slight reductions in opioid use in patients after undergoing a procedure, these reductions were mostly among people with no history of long term use (fewer than three prescriptions) or people who were prescribed weak opioids.

Many orthopaedic procedures (eg, knee and hip replacements) are often performed with the goal of reducing patient pain, which is often the indication for the procedure. However, reductions in opioid prescribing to patients after their procedure were lower than expected; one potential explanation is that osteoarthritis may affect more than the patient’s primary affected joint. Prolonged opioid use before surgery is also the greatest risk factor for long term use after surgery.^31^ Studies from Finland^9^ and Norway^10^ similarly found modest reductions in opioid prescribing among people with knee and hip arthroplasty, and also observed greater reduction in opioid use people with a hip procedure as compared those with knee procedure.

No standard definition of long term opioid use in routine data currently exists: definitions vary by study purpose, data availability, and investigator jurisdiction.^32^ We defined long term opioid use as three prescriptions in three months, which has been used previously in UK primary care data.^27^ However, other UK studies in primary care have defined it variably, including as three prescriptions in one year.^33^

### Strengths and limitations

In this study, we obtained data for a representative sample of about 43% of the English population.^34^ We could not compare our findings with studies involving people on waiting lists before the start of the covid-19 pandemic, because the Waiting List Minimum Dataset is a new repository established in response to the pandemic. However, we did compare opioid prescribing rates by individuals' time on the waiting list to better understand the effect of longer waits on opioid prescribing. Waits lasting for more than one year for trauma or orthopaedic procedures were extremely rare before the covid-19 pandemic, but were very common in our study cohort. Furthermore, the number of people on waiting lists remains substantial, with about one million more people on the waiting list for any treatment in July 2025 than in April 2022. More than 200 000 of these have been waiting for more than 52 weeks since referral.^20^ To our knowledge, this study is the first to use the person level waiting list data, and reports relevant findings on the potential harm to patients caused by long waits.

People with shorter waits may be systematically different from those waiting for longer periods, in ways that we could not ascertain (eg, type of procedure, health status); these factors may influence likelihood of opioid use. Further, the health condition for which the patient was areferred to the waiting list for and the treatment they received was not well recorded in the waiting list data. We performed sensitivity analyses among people with evidence of hip or knee procedures (or both), but we had to rely on linkage with hospital admissions data that can introduce a degree of error. ^35 36^

As we lacked information on the patients’ indication for prescribing, we had to exclude patients with other conditions that may be driving opioid prescribing, to understand how waiting list length influenced opioid prescribing specifically among people waiting for orthopaedic procedures. One potential reason for the limited reduction in opioid prescribing after a procedure is that some patients were using opioids to manage other conditions. Although we excluded people with a history of cancer, other chronic pain conditions may have driven some opioid prescribing and treatment of the referred condition would not have influenced opioid prescribing for other chronic pain conditions.

We did not have information on the dose of a single prescription. Although defining long term opioid use as three or more prescriptions in a three month period is an established approach,^27^ substantial variation in patient exposure to opioids may be present that we are not able to examine by counting prescription numbers. We have also classified opioids as strong, moderate, or weak, which does not reflect the exact dosage that patients were taking, and our analysis was restricted to primary care prescribing only (secondary care prescribing data is not relevant here, because long term opioid prescribing would only occur in primary care settings).

Our analysis was limited to people waiting to receive an orthopaedic procedure, to allow comparisons with cohorts in other international settings, with similar findings reported in Norway^10^ and Finland.^9^ However, generalising our findings to settings with different healthcare systems should be done cautiously: other factors may influence prescribing patterns.

### Policy implications

Since the rise in waiting times that began during the covid-19 pandemic, reducing time spent by patients on elective waiting lists has been a government priority.^37^ In mid-2024, the UK government pledged to clear the NHS waiting list backlog in five years, by increasing the number of available hospital appointments.^38^ Unnecessary exposure to opioid analgesia is among the many negative consequences of long wait times on patients, with well established risks to patients. The use of these medicines should, therefore, be minimised as much as possible. In 2023, NHS England issued instructions to healthcare professionals to improve standards and behaviours around opioid prescribing, highlighting the need for better use of data in preventing and reducing patient harm associated with opioids.^39^

Waiting lists are a key national priority, but we are unaware of any other studies using the new Waiting List Minimum Dataset’s patient level data for development of interventions and policy making.^40^ Our study demonstrates how using person level waiting list data, linked to general practice data within the OpenSAFELY platform, can be used to better understand opioid prescribing rates in a high risk population in line with key recommendations. We are developing tools to facilitate near real time audits and feedback in the context of rapidly changing pressures on the health service. These tools can include any measures supporting NHS England's ambition of safe opioid use and interventions to reduce the extended periods spent by patients on waiting lists, driven by the covid-19 pandemic.

### Future research

There may be heterogeneity in our findings that we have not identified, and could be addressed with additional research. Research into which patients are most likely to use opioids in the long term or experience other opioid related harms after inpatient procedures are needed, as well as investigations into reasons for non-discontinuation and how these changed during the covid-19 pandemic. An incidental finding was very high rates of prescribing of other analgesic medicines in addition to opioids, such as gabapentinoids and antidepressants, despite a lack of evidence for use in people with osteoarthritis^41^ and an increased risk of harms to the patient associated with co-prescribing.^42^ Current guidance on prescribing for people with chronic pain recommends a review of existing opioid prescribing to the patient.^43^ Medication reviews can be identified in primary care records, and future research could assess shared decision making practices around opioid prescribing before and after an individual’s referral to a waiting list.

The effect of longer wait times resulting from disruptions related to covid-19 is likely to extend beyond increased rates of analgesic prescribing. Delayed treatment can lead to muscle wasting caused by immobility, negatively affecting patient rehabilitation, in addition to the well documented psychological impacts and increased risk to patients of substance abuse caused by living with pain. ^44 45^ Longer waits are also associated with poorer patient outcomes for hip or knee arthroplasty, or joint replacements.^45^ The number of patients waiting for treatment has remained high since the study period.

The Waiting List Minimum Dataset linked to general practice records offers a new opportunity to assess changes on opioid prescribing since the covid-19 pandemic for patients who have been waiting since the pandemic began, and to understand how the NHS delivers care to these individuals.

### Conclusion

At a population level, while many factors may influence the rapid increase in the duration of and the number of people on a waiting list during the covid-19 pandemic, this population was smaller before the covid-19 pandemic. Understanding the resource and clinical needs of this new, large cohort is necessary to develop robust policy around reducing the waitlist and preventing future recurrence.^46^

Most people waiting for routine orthopaedic procedures waited for more than the standard 18 weeks to be admitted, and one in five waited for more than one year, which was very rare before the pandemic. We found that people on waiting lists experienced much longer wait times during the covid-19 pandemic, but we found no evidence that this longer wait influenced opioid prescribing patterns after their treatment.

## Supplementary material

10.1136/bmjmed-2025-001743 online supplemental file 1

## Data Availability

No data are available.
