# Supplementary material for: Opioid prescribing to people on orthopaedic waiting lists during the covid-19 pandemic in England: retrospective cohort study using linked electronic health record data in OpenSAFELY-TPP
Source: BMJ Med. 2025 Dec 10;4(1):e001743. doi: 10.1136/bmjmed-2025-001743 (PMC12699563; doi:10.1136/bmjmed-2025-001743)

Supplementary Files

Box 1: Glossary

Referral to treatment (RTT) pathway (time on waiting list): RTT pathways are consultant-led referrals for non-emergency services. It is initiated on the “clock start” date, and ends on the “clock stop” date.

Non-RTT pathway: Non-RTT pathways are non-consultant-led and planned care services such as physiotherapy, outpatient follow-ups, cancer surveillance, transplant follow ups, some diagnostics and chronic disease management.

Consultant-led: Treatment where a consultant has clinical responsibility. A consultant refers to a person contracted by a healthcare provider, has been appointed by a consultant appointment committee, and is a member of a Royal College or Faculty.

Clock start (waiting list referral date): The clock starts when the patient is referred to a consultant-led service where the patient will be assessed and, if appropriate, receive treatment; or an interface or referral management or assessment service, which may result in an onward referral to a consultant-led service.

Clock stop (waiting list end date): The clock can stop for treatment, which is the date when first definitive treatment starts; if the patient is admitted for treatment, the clock stop date is the date of admission. The clock doesn’t stop if:

- the patient is admitted for a diagnostic test / procedure only;
- the patient is admitted for pre-treatment;
- the patient is admitted for pre-op assessment only;
- the patient is admitted but doesn't receive the intended procedure.

The clock can also stop for non-treatment; for instance, if the patient enters active monitoring, the patient declines treatment, a clinical decision is made not to treat, or the patient dies.

First definitive treatment: An intervention intended to manage a patient’s disease. What constitutes first definitive treatment is based on clinical judgment in consultation with the patient.

Admitted pathway: RTT pathways that end with admission for an inpatient or day case procedure. These are sometimes referred to as inpatient waiting times.

Non-admitted pathway: Waits that end for reasons other than an inpatient or day case admission to hospital for treatment. These are sometimes referred to as outpatient waiting times. They include patients whose RTT waiting time clock either stopped for outpatient treatment or for non-treatment.

Adapted from: *Recording and reporting referral to treatment (RTT) waiting times for consultant-led elective care* (https://www.england.nhs.uk/statistics/statistical-work-areas/rtt-waiting-times/rtt-guidance/)

Supplementary Figure 1. Flow chart with study population inclusion and exclusion criteria

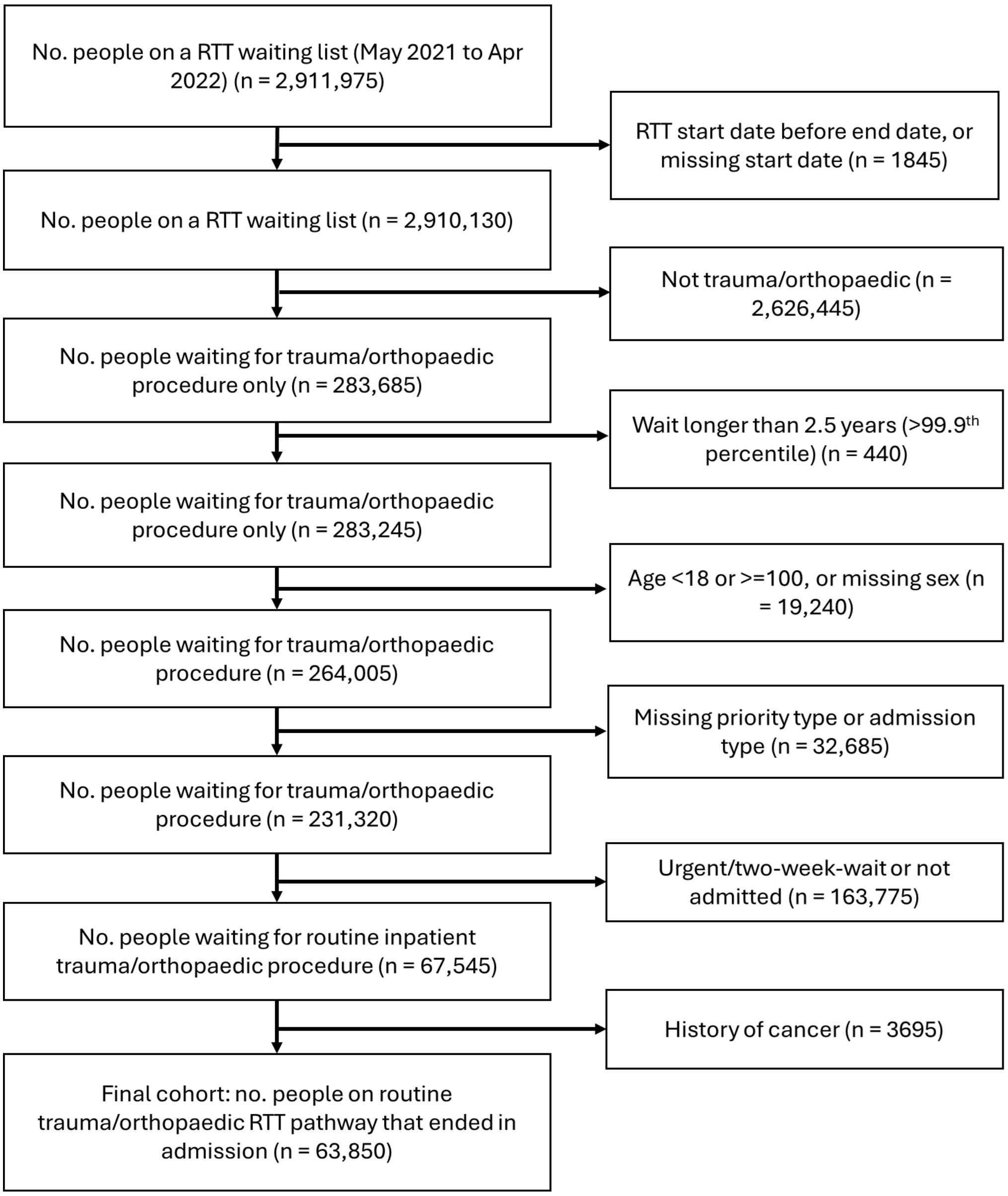

Supplementary Figure 2. Visualisation of study design for comparison of opioid prescribing before and after waiting list

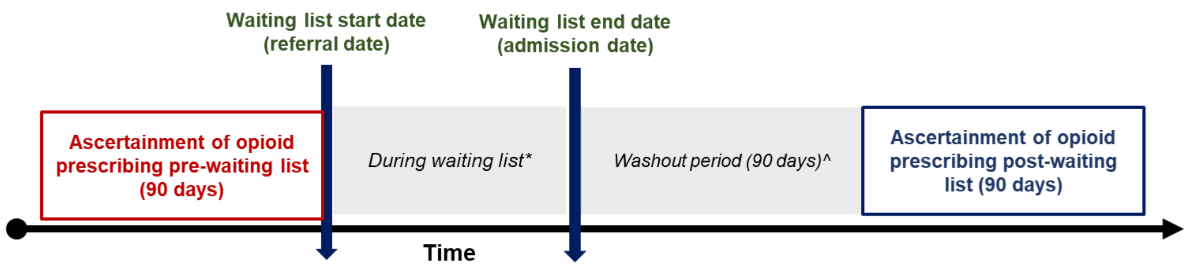

*Time on waiting list is variable for each person
^Washout period included to allow time for patients to discontinue opioids used to treat post-procedure pain in line with guidelines

Supplementary Table 1. List of non-trauma orthopaedic procedure HRG codes

**Hip procedures**	
HN12A	Very Major Hip Procedures for Non-Trauma with CC Score 10+
HN12B	Very Major Hip Procedures for Non-Trauma with CC Score 8-9
HN12C	Very Major Hip Procedures for Non-Trauma with CC Score 6-7
HN12D	Very Major Hip Procedures for Non-Trauma with CC Score 4-5
HN12E	Very Major Hip Procedures for Non-Trauma with CC Score 2-3
HN12F	Very Major Hip Procedures for Non-Trauma with CC Score 0-1
HN13A	Major Hip Procedures for Non-Trauma, 19 years and over, with CC Score 10+
HN13B	Major Hip Procedures for Non-Trauma, 19 years and over, with CC Score 6-9
HN13C	Major Hip Procedures for Non-Trauma, 19 years and over, with CC Score 4-5
HN13D	Major Hip Procedures for Non-Trauma, 19 years and over, with CC Score 2-3
HN13E	Major Hip Procedures for Non-Trauma, 19 years and over, with CC Score 1
HN13F	Major Hip Procedures for Non-Trauma, 19 years and over, with CC Score 0
HN13G	Major Hip Procedures for Non-Trauma, 18 years and under, with CC Score 1+
HN13H	Major Hip Procedures for Non-Trauma, 18 years and under, with CC Score 0
HN14A	Intermediate Hip Procedures for Non-Trauma, 19 years and over, with CC Score 6+
HN14B	Intermediate Hip Procedures for Non-Trauma, 19 years and over, with CC Score 4-5
HN14C	Intermediate Hip Procedures for Non-Trauma, 19 years and over, with CC Score 2-3
HN14D	Intermediate Hip Procedures for Non-Trauma, 19 years and over, with CC Score 1
HN14E	Intermediate Hip Procedures for Non-Trauma, 19 years and over, with CC Score 0
HN14F	Intermediate Hip Procedures for Non-Trauma, between 6 and 18 years, with CC Score 1+
HN14G	Intermediate Hip Procedures for Non-Trauma, between 6 and 18 years, with CC Score 0
HN14H	Intermediate Hip Procedures for Non-Trauma, 5 years and under
HN15A	Minor Hip Procedures for Non-Trauma, 19 years and over
HN15B	Minor Hip Procedures for Non-Trauma, 18 years and under
HN16A	Minimal Hip Procedures, 19 years and over
HN16B	Minimal Hip Procedures, between 6 and 18 years

HN16C	Minimal Hip Procedures, 5 years and under
**Knee procedures**	
HN22A	Very Major Knee Procedures for Non-Trauma with CC Score 8+
HN22B	Very Major Knee Procedures for Non-Trauma with CC Score 6-7
HN22C	Very Major Knee Procedures for Non-Trauma with CC Score 4-5
HN22D	Very Major Knee Procedures for Non-Trauma with CC Score 2-3
HN22E	Very Major Knee Procedures for Non-Trauma with CC Score 0-1
HN23A	Major Knee Procedures for Non-Trauma, 19 years and over, with CC Score 4+
HN23B	Major Knee Procedures for Non-Trauma, 19 years and over, with CC Score 2-3
HN23C	Major Knee Procedures for Non-Trauma, 19 years and over, with CC Score 0-1
HN23D	Major Knee Procedures for Non-Trauma, 18 years and under, with CC Score 1+
HN23E	Major Knee Procedures for Non-Trauma, 18 years and under, with CC Score 0
HN24A	Intermediate Knee Procedures for Non-Trauma, 19 years and over, with CC Score 4+
HN24B	Intermediate Knee Procedures for Non-Trauma, 19 years and over, with CC Score 2-3
HN24C	Intermediate Knee Procedures for Non-Trauma, 19 years and over, with CC Score 0-1
HN24D	Intermediate Knee Procedures for Non-Trauma, between 6 and 18 years, with CC Score 1+
HN24E	Intermediate Knee Procedures for Non-Trauma, between 6 and 18 years, with CC Score 0
HN24F	Intermediate Knee Procedures for Non-Trauma, 5 years and under
HN25A	Minor Knee Procedures for Non-Trauma, 19 years and over
HN25B	Minor Knee Procedures for Non-Trauma, 18 years and under
HN26A	Minimal Knee Procedures, 19 years and over
HN26B	Minimal Knee Procedures, between 6 and 18 years
HN26C	Minimal Knee Procedures, 5 years and under

Supplementary Figure 3. Distribution of waiting list start date (referral date) and end date (admission date) (n = 63,850)

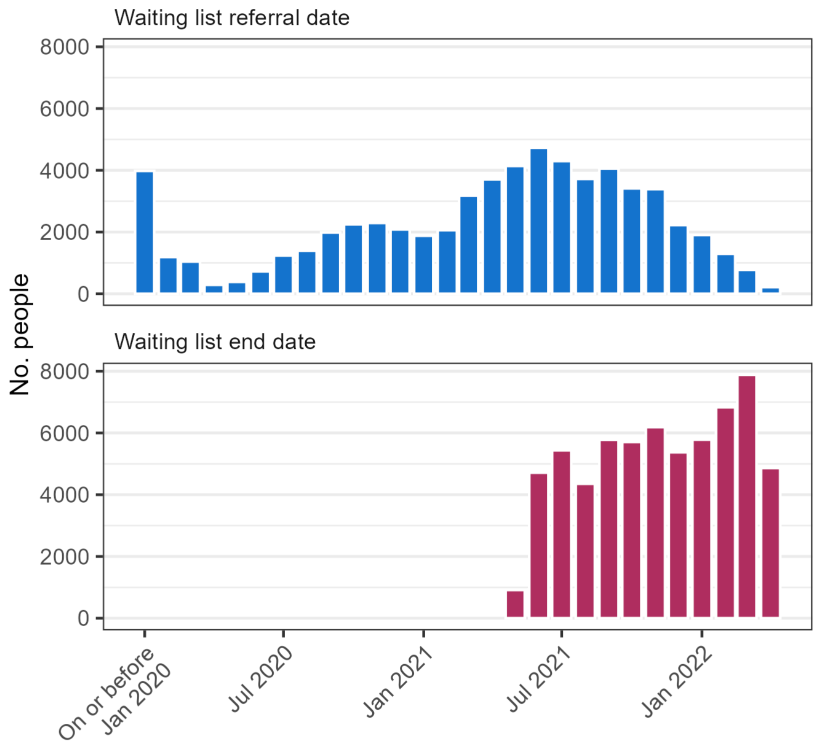

Supplementary Table 2. Characteristics of people with recorded osteoarthritis diagnosis, and who received a hip or knee procedure for sensitivity analyses

	**Osteoarthritis diagnosis**	**Hip procedure**	**Knee procedure**
	**n (%)**	**n (%)**	**n (%)**
**Total**	24,255 (100.0)	10,160 (100.0)	14,975 (100,0)
**Time on waiting list**			
<=18 weeks	8370 (34.5)	3220 (31.7)	4755 (31.8)
19-52 weeks	10275 (42.4)	4655 (45.8)	6475 (43.2)
>52 weeks	5610 (23.1)	2285 (22.5)	3745 (25.0)
**Age**			
18-39	430 (1.8)	505 (5.0)	2390 (16.0)
40-49	1380 (5.7)	650 (6.4)	1515 (10.1)
50-59	4855 (20)	1720 (16.9)	2890 (19.3)
60-69	7305 (30.1)	2780 (27.4)	3605 (24.1)
70-79	7620 (31.4)	3285 (32.3)	3565 (23.8)
80+	2670 (11.0)	1215 (12.0)	1005 (6.7)
**Sex**			
Female	14355 (59.2)	6105 (60.1)	7375 (49.2)
Male	9905 (40.8)	4055 (39.9)	7595 (50.7)
**IMD decile**			
1 (most deprived)	1895 (7.8)	655 (6.4)	1330 (8.9)
2	2205 (9.1)	845 (8.3)	1420 (9.5)
3	2230 (9.2)	865 (8.5)	1365 (9.1)
4	2360 (9.7)	975 (9.6)	1480 (9.9)
5	2540 (10.5)	1060 (10.4)	1560 (10.4)
6	2875 (11.9)	1260 (12.4)	1720 (11.5)
7	2690 (11.1)	1230 (12.1)	1635 (10.9)
8	2515 (10.4)	1110 (10.9)	1490 (9.9)
9	2470 (10.2)	1085 (10.7)	1480 (9.9)
10 (least deprived)	2075 (8.6)	900 (8.9)	1210 (8.1)
Missing	405 (1.7)	175 (1.7)	285 (1.9)
**Ethnicity**			
White	18675 (77)	7790 (76.7)	10825 (72.3)
Black	525 (2.2)	60 (0.6)	500 (3.3)
South Asian	160 (0.7)	50 (0.5)	145 (1.0)
Mixed	80 (0.3)	30 (0.3)	85 (0.6)
Other	75 (0.3)	30 (0.3)	85 (0.6)
Unknown	4740 (19.5)	2200 (21.7)	3330 (22.2)
**Conditions recorded in primary care in past 5 years**			
Anxiety (symptoms or diagnosis)	2755 (11.4)	925 (9.1)	1595 (10.7)
Cardiac disease	3575 (14.7)	1325 (13)	1570 (10.5)
Chronic kidney disease	1200 (4.9)	465 (4.6)	500 (3.3)
Chronic respiratory disease	1360 (5.6)	540 (5.3)	490 (3.3)
Depression (symptoms or diagnosis)	3055 (12.6)	1025 (10.1)	1725 (11.5)
Diabetes	2725 (11.2)	890 (8.8)	1330 (8.9)
Osteoarthritis	24255 (100.0)	6050 (59.5)	7015 (46.8)
Rheumatoid arthritis	555 (2.3)	170 (1.7)	290 (1.9)
**Prescribing in 3 months prior to waiting list**			
Antidepressants			
≥1 prescription	7055 (29.1)	2515 (24.8)	3290 (22)
≥3 prescriptions	4225 (17.4)	1405 (13.8)	1815 (12.1)

Amitriptyline/duloxetine			
≥1 prescription	3455 (14.2)	1250 (12.3)	1335 (8.9)
≥3 prescriptions	1760 (7.3)	605 (6.0)	670 (4.5)
Gabapentinoids			
≥1 prescription	995 (9.8)	1125 (7.5)	995 (9.8)
≥3 prescriptions	625 (6.2)	740 (4.9)	625 (6.2)
NSAIDs			
≥1 prescription	4780 (19.7)	2100 (20.7)	2540 (17.0)
≥3 prescriptions	1705 (7.0)	725 (7.1)	730 (4.9)
**Died in 6 months following waiting list end date**	140 (0.6)	45 (0.4)	35 (0.2)

Supplementary Table 3. Opioid prescribing before and after time on waiting list among groups for sensitivity analyses.“Pre- waiting list” is 3 months prior to referral date, and “Post-waiting list” is months 4-6 after waiting list end date.

	≥1 prescription		≥3 more prescriptions	
	**Pre-waiting list**	**Post-waiting list***	**Pre-waiting list**	**Post-waiting list***
**Osteoarthritis diagnosis**				
Any opioid	10,345 (42.7)	8,065 (35)	5,160 (21.3)	4,395 (19.1)
Immediate-release opioid	9,515 (39.2)	7,270 (31.5)	4,215 (17.4)	3,550 (15.4)
Modified-release opioid	1,750 (7.2)	1,545 (6.7)	1,210 (5.0)	1,135 (4.9)
Weak opioid	7,585 (31.3)	5,565 (24.1)	2,950 (12.2)	2,475 (10.7)
Moderate opioid	2,140 (8.8)	1,660 (7.2)	1,050 (4.3)	915 (4.0)
Strong opioid	1,945 (8)	1,735 (7.5)	1,265 (5.2)	1,195 (5.2)
**Knee procedure**				
Any opioid	4,860 (32.5)	3,890 (27.3)	2,210 (14.8)	1,980 (13.9)
Immediate-release opioid	4,505 (30.1)	3,560 (25)	1,820 (12.2)	1,660 (11.7)
Modified-release opioid	695 (4.6)	595 (4.2)	470 (3.1)	435 (3.1)
Weak opioid	3,660 (24.4)	2,785 (19.6)	1,270 (8.5)	1,185 (8.3)
Moderate opioid	1,015 (6.8)	820 (5.8)	485 (3.2)	420 (3.0)
Strong opioid	750 (5.0)	690 (4.8)	485 (3.2)	465 (3.3)
**Hip procedure**				
Any opioid	4,525 (44.5)	2,925 (30.2)	2,180 (21.5)	1,530 (15.8)
Immediate-release opioid	4,225 (41.6)	2,630 (27.2)	1,845 (18.2)	1,220 (12.6)
Modified-release opioid	665 (6.5)	550 (5.7)	425 (4.2)	400 (4.1)
Weak opioid	3,515 (34.6)	2,090 (21.6)	1,365 (13.4)	880 (9.1)
Moderate opioid	835 (8.2)	530 (5.5)	390 (3.8)	295 (3.0)
Strong opioid	765 (7.5)	610 (6.3)	470 (4.6)	415 (4.3)

*People with complete follow-up

Supplementary Figure 4. Number of opioid prescriptions per week in the 26 weeks prior to waiting list referral date, during the waiting list, and 52 weeks after waiting list end date among people with a **recorded diagnosis of osteoarthritis only**. Dots are observed values, solid lines are values predicted from loess regression model. Shaded area is 3 months post-admission.

Legend: During the waiting list period, the denominator includes everyone who was still on the waiting list at the end of each week. During the waiting list period and post-waiting list periods, the denominator excludes people who died or who deregistered from their general practice.

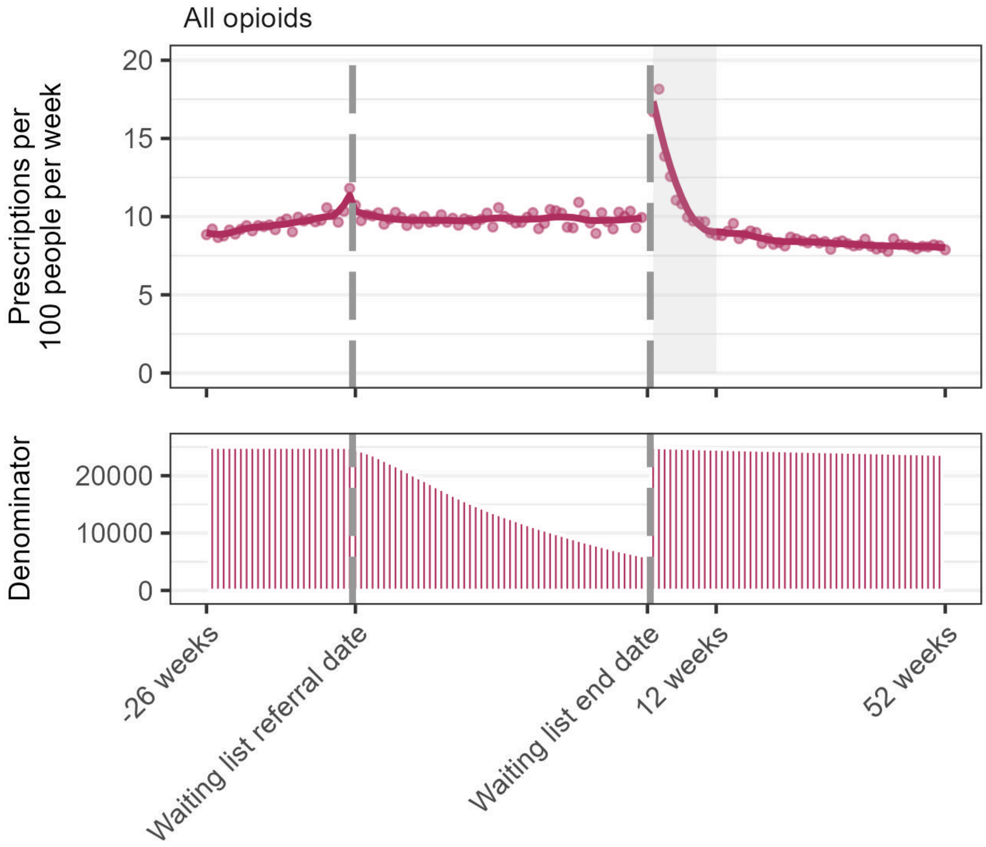

Supplementary Figure 5. Number of opioid prescriptions per week in the 26 weeks prior to waiting list referral date, during the waiting list, and 52 weeks after waiting list end date among people who had a **hip procedure**. Dots are observed values, solid lines are values predicted from loess regression model. Shaded area is 3 months post-admission.

Legend: During the waiting list period, the denominator includes everyone who was still on the waiting list at the end of each week. During the waiting list period and post-waiting list periods, the denominator excludes people who died or who deregistered from their general practice.

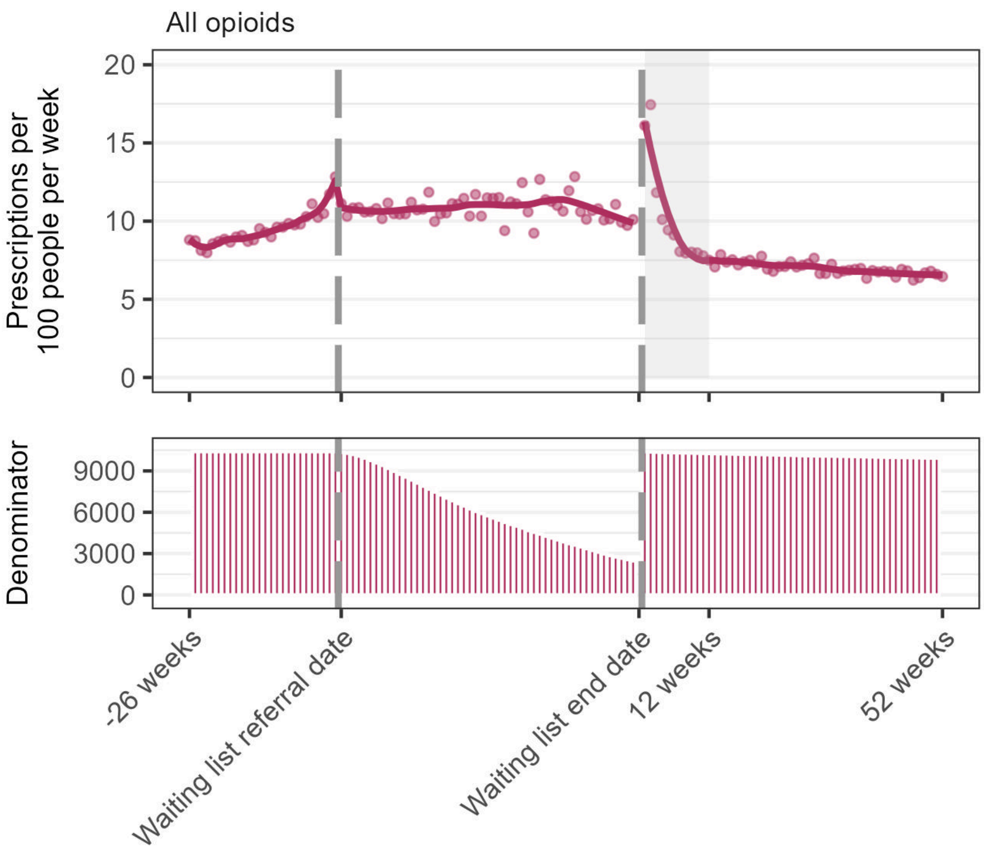

Supplementary Figure 6. Number of opioid prescriptions per week in the 26 weeks prior to waiting list referral date, during the waiting list, and 52 weeks after waiting list end date among people who had a **knee procedure**. Dots are observed values, solid lines are values predicted from loess regression model. Shaded area is 3 months post-admission.

Legend: During the waiting list period, the denominator includes everyone who was still on the waiting list at the end of each week. During the waiting list period and post-waiting list periods, the denominator excludes people who died or who deregistered from their general practice.

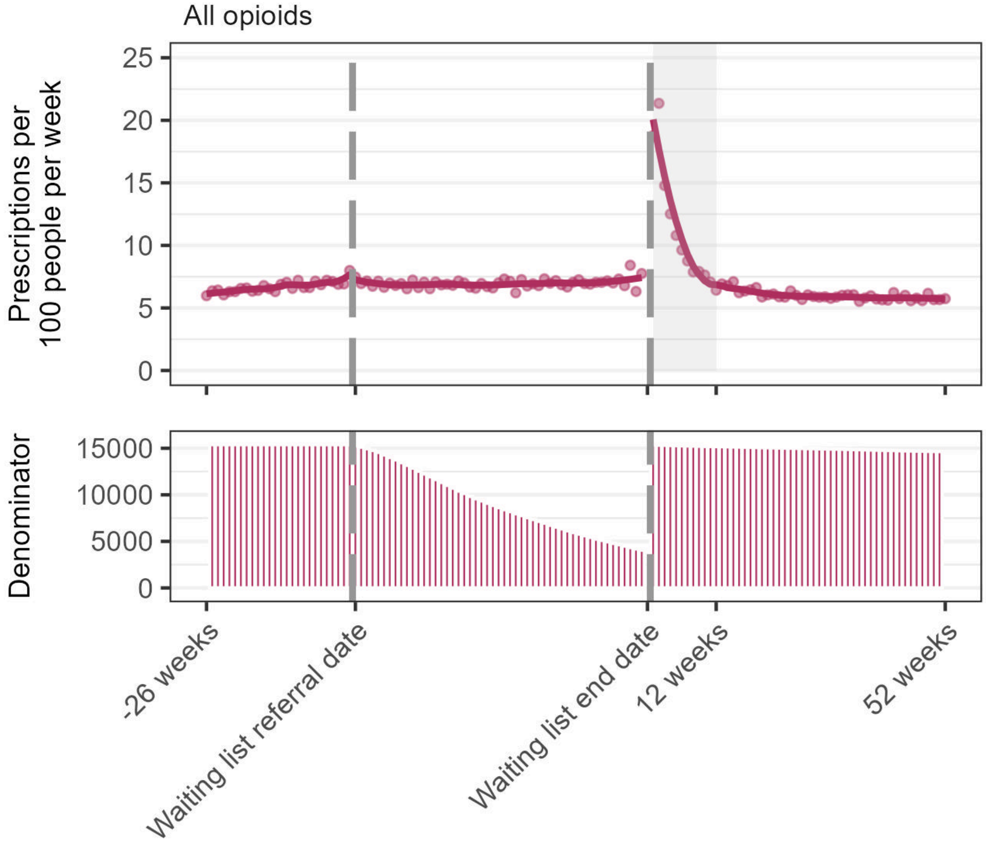

Supplement: online supplemental file 1 [file bmjmed-4-1-s001.pdf]
