# Supplementary material for: Opioid prescribing to people on orthopaedic waiting lists during the covid-19 pandemic in England: retrospective cohort study using linked electronic health record data in OpenSAFELY-TPP
Source: BMJ Med. 2025 Dec 10;4(1):e001743. doi: 10.1136/bmjmed-2025-001743 (PMC12699563; doi:10.1136/bmjmed-2025-001743)
Supplement: Supplementary file 2 [file bmjmed-2025-001743.reviewer_comments.pdf]

PEER REVIEW HISTORY

BMJ Medicine publishes all reviews undertaken for accepted manuscripts. Reviewers are asked to complete a checklist review form and are provided with free text boxes to elaborate on their assessment. These free text comments are reproduced below.

ARTICLE DETAILS

**TITLE (PROVISIONAL)**	Opioid prescribing to people on orthopaedic waiting lists during the COVID-19 pandemic in England: a study using OpenSAFELY-TPP
**AUTHORS**	Higgins, Rose; Smith, Rebecca; Dillingham, Iain; Quinlan, Jane; Speed, Victoria; Curtis, Helen; Wood, Christopher; Wiedemann, Milan; Jani, Meghna; Bacon, Sebastian; Mehrkar, Amir; Goldacre, Ben; Collective, The OpenSAFELY Collective; MacKenna, Brian; Schaffer, Andrea

VERSION 1 - REVIEW

**REVIEWER NAME**	Schofield, Anne-Marie
**REVIEWER AFFILIATION**	Hôpital Lariboisière
**REVIEWER CONFLICT OF INTEREST**	None
**AI DISCLOSURE**	No
**DATE REVIEW RETURNED**	01-Jul-2025

**GENERAL COMMENTS**	First of all I would like to thank you for considering me as a reviewer for this manuscript which is clearly the result of an extensive work.<br><br>The explanations provided to understand the figures, and the choice of outcomes measures seem complex. It might be helpful to simplify and clarify some aspects particularly in the introduction by providing a brief overview of the UK healthcare system for readers who practice outside the UK.<br><br>I also have a few questions regarding the data : were the medical prescriptions cross-checked with pharmacy records ? Would it be possible to retrieve a clinical pain score from the patients ?<br><br>The sensitivity analysis focusing on hip and knee arthroplasties is definitely relevant. However do we know which specific procedures patients were awaiting ? A patient waiting for an anterior cruciate ligament reconstruction is likely to have a different pain and functional level than someone scheduled for a total hip arthroplasty or a scaphoid non-union surgery.<br><br>Although careful attention was given to exclude patients awaiting an urgent procedure, do we know how many of these patients were likely to be included in the final analysis ?<br><br>The clinical question, whether the Covid 19 pandemic and increased waiting times impacted opiod prescribing has been adressed. However I wonder what implications these findings will have in practice especially considering the exceptional nature of the pandemic context.<br>Given that the WLDMS was established during the Covid-19 period, it would be particularly relevant and arguably necessary to identify a

	suitable pre-pandemic reference population in order to assess more rigorously the impact of prolonged preoperative waiting times on opioid prescriptions. It would considerably strengthen the validity and interpretability of these findings beyond the exceptional context of the pandemic.

**REVIEWER NAME**	QIN, ZONGSHI
**REVIEWER AFFILIATION**	Peking University Clinical Research Institute
**REVIEWER CONFLICT OF INTEREST**	None
**AI DISCLOSURE**	No
**DATE REVIEW RETURNED**	03-Jul-2025

**GENERAL COMMENTS**	The authors used data from a retrospective cohort study to understand the association between opioid prescription wait times and opioid usage. Although the data were collected in England, I think the conclusions might be generalised to other countries. This is a well-structured manuscript with comprehensive data analysis and supplemental documents. I agree with most of the conclusions based on the current study design and dataset. As the COVID-19 pandemic has ended for 2 years, I do not want to waste any time slowing down the publication of this study.

**REVIEWER NAME**	van Duinen, Gabriel
**REVIEWER AFFILIATION**	
**REVIEWER CONFLICT OF INTEREST**	None
**AI DISCLOSURE**	No
**DATE REVIEW RETURNED**	14-Jul-2025

**GENERAL COMMENTS**	Thank you very much for asking me to review Higgins et al. (2025) Opioid prescribing to people on orthopaedic waiting lists during the COVID-19 pandemic in England: a study using OpenSAFELY-TPP.<br><br>General Comment<br><br>Overall, this paper was well written and understandable by a lay audience. It presents interesting findings (and associated policy implications) around the impact of orthopaedic waiting lists and opioid usage which will likely generate a wide amount of interest from the general public if published.<br><br>One area where I did have more questions after reading this paper was around the reasons for non-discontinuation of opioid treatment after surgery. This is outside of the scope of this research but it is an area which may generate subsequent discussions if this paper was published. If there is an accompanying editorial to this article, I would suggest that the existing research on opioid use/dependence would be useful to discuss in it.<br><br>Patient and Public Engagement<br><br>The authors should be commended for their very good patient and public engagement. This engagement has occurred throughout all

	parts of the research lifecycle with a variety of engagement forms used including a mix of long-term (e.g., patient members of advisory committees) and short-term (e.g., dissemination of findings) engagement.<br><br>Thank you for asking me to review this paper. I remain available to review a revised version if required.

**REVIEWER NAME**	Davies, Neil
**REVIEWER AFFILIATION**	UCL, Division of Psychiatry and Department of Statistical Science
**REVIEWER CONFLICT OF INTEREST**	None
**AI DISCLOSURE**	No
**DATE REVIEW RETURNED**	05-Aug-2025

**GENERAL COMMENTS**	bmjmed-2025-001743 Opioid prescribing to people on orthopaedic waiting lists during the COVID-19 pandemic in England: a study using OpenSAFELY-TPP<br><br>This is a solid paper that describes rates of opioid prescription among people on orthopaedic waiting lists in England post pandemic. The paper is very well written and the analysis is credible and thoughtful. The authors limit themselves to describing differences in prescription rates by time on waiting lists and are very conservative about their interpretation.<br><br>The biggest limitation of the paper is the very restricted number of years of data that are covered. It's not immediately obvious why these years were chosen, or why the analysis hasn't been updated to reflect a more recent extract of data, aside from the fact that this is the data that was available at the time. I do not believe this limitation precludes publication, and understand why the authors haven't updated the paper. But it makes it quite hard to interpret any of their findings around the changes to prescribing pre- and post-pandemic and the implications for patient care and opioid treatment rates.<br>As a result of the sampling frame, the policy implications are not clear. We do not know whether opioid use has increased as a result of the increased waiting times relative to pre-pandemic. We do not know whether opioid use has remained elevated or has returned to pre-pandemic levels. As a result, despite using national data, the findings are not very informative – they represent a very limited cross-section of time post-pandemic that is increasingly historical. This isn't a statistical flaw per se, and there is likely little the authors can do to overcome this, short of rerunning a much more comprehensive analysis on a longer time frame. However, it does limit the impact of the paper.<br><br>Statistically, the paper is sound, well-conducted, and worthy of publication.<br><br>Minor<br>"Opioid *prescriptions* among eligible patients during and after referral to the waiting list and discharge from treatment."

VERSION 1 – AUTHOR RESPONSE

Reviewer: 1

Dr. Anne-Marie Schofield, Hôpital Lariboisière

Comments to the Author

Dear Author,

First of all I would like to thank you for considering me as a reviewer for this manuscript which is clearly the result of an extensive work.

Thank you for your consideration and comments.

The explanations provided to understand the figures, and the choice of outcomes measures seem complex. It might be helpful to simplify and clarify some aspects particularly in the introduction by providing a brief overview of the UK healthcare system for readers who practice outside the UK.

To clarify the context for international readers we have added the following context:

Methods>Context, page 4: *"In England, patients referred by the National Health Service (NHS) for a physical or mental health condition have a right to start non-urgent, consultant-led treatment within 18 weeks. A patient's waiting time starts at the date of referral to treatment, and ends when a clinician decides no treatment is necessary, if the patient declines treatment, or when treatment for the referred condition starts. Primary care is the first point of contact with the health service for most non-urgent care, and as such prescribing for patients being referred via their primary care provider will be captured in the primary care data."*

I also have a few questions regarding the data : were the medical prescriptions cross-checked with pharmacy records ? Would it be possible to retrieve a clinical pain score from the patients ?

These were not cross checked with pharmacy records as these are not available, however these data have been used for many different studies of medicines and are considered quite accurate as a recorded prescription in primary care is necessary for getting a prescription. They also compare well with the dispensing data at practice level (available through our tool OpenPrescribing).

It's unclear how well-captured pain scores are in primary care for this population. However, we are unable to retrieve additional data as this study received legal permission under COVID-19 Control of Patient Information (COPI) notice. Briefly, this notice provides access to patient records for the purposes of urgent analyses related to the COVID-19 pandemic. The COPI notice expired June 2023, and the legal basis of the OpenSAFELY COVID-19 Service transitioned to a Direction under which OpenSAFELY is required to respect Type 1 Opt-outs (T1OO) by making the data of anyone who has registered a T1OO unavailable to users. All projects that were approved in OpenSAFELY under the COPI notice were unable to continue to process data without new approvals being granted under the new legal basis. Unfortunately, as we received these comments after the expiration of the COPI notice, we are unable to run any further analyses in response to reviewer comments.

The sensitivity analysis focusing on hip and knee arthroplasties is definitely relevant. However do we know which specific procedures patients were awaiting ? A patient waiting for an anterior cruciate ligament reconstruction is likely to have a different pain and functional level than someone scheduled for a total hip arthroplasty or a scaphoid non-union surgery.

While the reviewer is correct that patients may be waiting for a wide range of different procedures, the purpose of this study was not to identify predictors of opioid use. As there is a large number of potential procedures, selecting and coding all individual procedures would not be expedient and would result in small counts for each procedure group, making interpretation of results difficult. Further, the accuracy of procedure coding is uncertain. Therefore, for this study we utilised the higher level grouping of Healthcare Resource Groups (HRGs) to identify groups of procedures which identifies groups of people treated with similar procedures, while ensuring adequate counts for subgroup analyses. A clinician reviewed the HRGs used in the study to be inclusive of activity within the scope of this study.

Although careful attention was given to exclude patients awaiting an urgent procedure, do we know how many of these patients were likely to be included in the final analysis ?

By definition the restriction in the analysis to elective care excludes urgent care and there is no established way to identify patients actually receiving urgent care and thus misclassified. While some misclassification is always possible, we have no reason to suspect it is meaningful in this dataset and therefore is unlikely to have substantial impact on our results.

The clinical question, whether the Covid 19 pandemic and increased waiting times impacted opiod prescribing has been adressed. However I wonder what implications these findings will have in practice especially considering the exceptional nature of the pandemic context.

As the study period did not include the acute lockdown phase of the pandemic we feel these results are generalisable to a post-pandemic period. As the waiting list backlog and waiting times have not reduced significantly since our study period (https://www.bma.org.uk/advice-and-support/nhs-delivery-and-workforce/pressures/nhs-backlog-data-analysis), the results we found are still relevant in terms of clinical considerations to prescribing during periods where long waiting times for treatment are highly prevalent.

We have added context around the ongoing waiting list backlog:

Discussion>Strength and Limitations: "*Furthermore, waiting lists remain significant to date, with approximately 1 million more people on the waiting list in July 2025 than in April 2022 and over 200,000 people who have been waiting more than 52 weeks since referral[48].*"

Given that the WLDMS was established during the Covid-19 period, it would be particularly relevant and arguably necessary to identify a suitable pre-pandemic reference population in order to assess more rigorously the impact of prolonged preoperative waiting times on opioid prescriptions. It would considerably strengthen the validity and interpretability of these findings beyond the exceptional context of the pandemic.

We agree that it would be informative to compare our results during the pandemic to pre-pandemic prescribing. However, the waiting list backlog and waiting times have reduced little since our study period, and it's still important to understand the current situation, regardless of how this compares to pre-pandemic. Ongoing monitoring of waiting times and their impact on opioid prescribing and how these change over time into the future is warranted.

We have added this as a potential for future research:

Discussion>Future research, page 16: *"The number of patients waiting for treatment has remained high since the study period. Future research could assess if there have been changes since the pandemic on opioid prescribing for patients waiting since the pandemic."*

This study is novel as it is the first of its kind to utilise the person-level waiting list data, and is a demonstration of the potential of this dataset in future research more generally.

Reviewer: 2

Dr. ZONGSHI QIN, Peking University Clinical Research Institute

Comments to the Author

The authors used data from a retrospective cohort study to understand the association between opioid prescription wait times and opioid usage. Although the data were collected in England, I think the conclusions might be generalised to other countries.

We agree, and in our 'Findings in context' section in the Discussion refer to two studies from Finland and Norway which showed similar modest reductions in postoperative opioids prescribing in knee and hip arthroplasty.

To further emphasise this point, we have added a sentence regarding the generalisability to other countries:

Discussion>Strengths and limitations, page 15: *"We have restricted our analysis to people waiting to receive an orthopaedic procedure allowing comparisons with cohorts in other international settings, with similar findings reported in Norway and Finland (see above). However caution should be taken in generalising to settings with different healthcare systems, where other factors may influence prescribing behaviours."*

This is a well-structured manuscript with comprehensive data analysis and supplemental documents. I agree with most of the conclusions based on the current study design and dataset. As the COVID-19 pandemic has ended for 2 years, I do not want to waste any time slowing down the publication of this study.

Thank you for your time and consideration.

Reviewer: 3

Mr. Gabriel van Duinen

Comments to the Author

Dear Editor,

Thank you very much for asking me to review Higgins et al. (2025) Opioid prescribing to people on orthopaedic waiting lists during the COVID-19 pandemic in England: a study using OpenSAFELY-TPP.

Thank you for your time and consideration.

General Comment

Overall, this paper was well written and understandable by a lay audience. It presents interesting findings (and associated policy implications) around the impact of orthopaedic waiting lists and opioid usage which will likely generate a wide amount of interest from the general public if published.

One area where I did have more questions after reading this paper was around the reasons for non-discontinuation of opioid treatment after surgery. This is outside of the scope of this research but it is an area which may generate subsequent discussions if this paper was

published. If there is an accompanying editorial to this article, I would suggest that the existing research on opioid use/dependence would be useful to discuss in it.

We agree with the suggestion of future work. That work would be helped by ongoing access to the WLMDS data used in this study. We have added a line to reflect the suggestion of this future work:

Discussion>Future research: "*More research into which patients are most likely to continue with long-term opioid use or experience other opioid-related harms after*

inpatient procedures, reasons for non-discontinuation and how this changed during the pandemic, is needed"

Patient and Public Engagement

The authors should be commended for their very good patient and public engagement. This engagement has occurred throughout all parts of the research lifecycle with a variety of engagement forms used including a mix of long-term (e.g., patient members of advisory committees) and short-term (e.g., dissemination of findings) engagement.

Thank you for asking me to review this paper. I remain available to review a revised version if required.

Gabriel Van Duinen

Patient/Public Reviewer

Reviewer: 4

Prof. Neil Davies, UCL

Comments to the Author

bmjmed-2025-001743 Opioid prescribing to people on orthopaedic waiting lists during the COVID-19 pandemic in England: a study using OpenSAFELY-TPP

This is a solid paper that describes rates of opioid prescription among people on orthopaedic waiting lists in England post pandemic. The paper is very well written and the analysis is credible and thoughtful. The authors limit themselves to describing differences in prescription rates by time on waiting lists and are very conservative about their interpretation.

Thank you for your time and consideration.

The biggest limitation of the paper is the very restricted number of years of data that are covered. It's not immediately obvious why these years were chosen, or why the analysis hasn't been updated to reflect a more recent extract of data, aside from the fact that this is the data that was available at the time. I do not believe this limitation precludes publication, and understand why the authors haven't updated the paper. But it makes it quite hard to interpret any of their findings around the changes to prescribing pre- and post-pandemic and the implications for patient care and opioid treatment rates.

As a result of the sampling frame, the policy implications are not clear. We do not know whether opioid use has increased as a result of the increased waiting times relative to pre-pandemic. We do not know whether opioid use has remained elevated or has returned to pre-

pandemic levels. As a result, despite using national data, the findings are not very informative – they represent a very limited cross-section of time post-pandemic that is increasingly historical. This isn't a statistical flaw per se, and

there is likely little the authors can do to overcome this, short of rerunning a much more comprehensive analysis on a longer time frame. However, it does limit the impact of the paper.

The person-level waiting list dataset was established during the pandemic period, and only a one off data feed for the study period obtained, hence the limited time period of the study. We believe this is the first paper using this data for research, and as such is a demonstration of the power of linked patient level datasets in understanding the profile of patients on waiting lists in England. In spite of this limitation, this work can inform current understanding; for example, waiting list sizes remain large (over 1 million more people waiting in July 2025 than in April 2022), and waits remain long (in July 2025 there were over 200,000 people who had waited for more than 52 weeks for treatment), having changed little since our study period (according to data published by NHSE here). Thus, it's still important to understand the current situation, regardless of how this compares to pre-pandemic. Long wait times and waiting list backlogs are currently a very important policy issue. As a result, we believe our findings are still highly relevant, and demonstrate the value of having ongoing access to such patient-level data.

We have added a paragraph describing the limitations of our access to the data, as well as the relevance to the current waiting list size and length of waits:

Discussion>Strengths and limitations:"*Furthermore, waiting lists remain significant to date, with approximately 1 million more people on the waiting list in July 2025 than in April 2022 and over 200,000 people who have been waiting more than 52 weeks since referral[REF]. This study represents the first published analysis using the person-level waiting list data, and findings on the potential harm due to long waits and the utility of this data are still relevant in the current landscape.*"

Statistically, the paper is sound, well-conducted, and worthy of publication.

Minor

"Opioid *prescriptions* among eligible patients during and after referral to the waiting list and discharge from treatment."

Corrected.

VERSION 2 – REVIEW

**REVIEWER NAME**	Schofield, Anne-Marie
**REVIEWER AFFILIATION**	Hôpital Lariboisière
**REVIEWER CONFLICT OF INTEREST**	None
**AI DISCLOSURE**	No
**DATE REVIEW RETURNED**	08-Oct-2025

**GENERAL COMMENTS**	Thank you for the revision of this paper<br>You have addressed all my comments<br>I do not have further more comments to make.
